# Application of Carbon Nanotubes from Waste Plastics As Filler to Epoxy Resin Composite

**DOI:** 10.1021/acssuschemeng.1c07776

**Published:** 2022-02-01

**Authors:** Yuanyuan Wang, Ning Cai, Haiping Yang, Chunfei Wu

**Affiliations:** † School of Chemistry and Chemical Engineering, Queen’s University Belfast, Belfast, BT7 1NN, United Kingdom; ‡ State Key Laboratory of Coal Combustion, School of Energy and Power Engineering, Huazhong University of Science and Technology, Wuhan, 430074, People’s Republic of China

**Keywords:** Carbon nanotubes, Waste plastic, Epoxy resin, Mechanical properties, Polymer composites

## Abstract

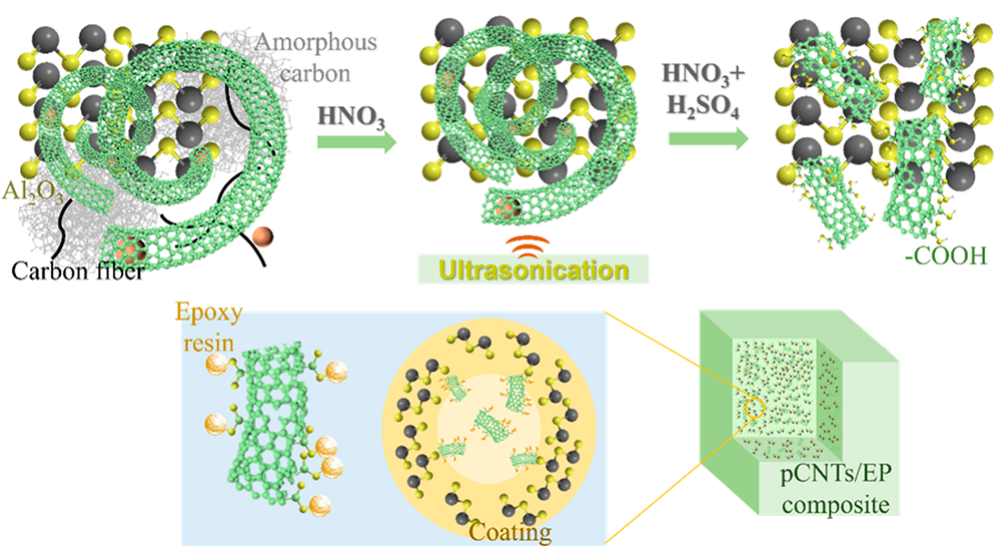

Carbon nanotubes (CNTs) are promising nanofillers to enhance the mechanical performance of polymers. Through catalytic conversion, waste plastics can be converted into CNTs, which could be an alternative to commercial CNTs (cCNTs). Exploring a practical application of waste-plastic-derived CNTs will largely promote the technology development related to waste plastic management and CNT production. In this work, CNTs produced from plastics, named pCNTs, were applied as fillers to epoxy resin (EP), while commercial CNTs (cCNTs) were used as a reference. The carboxyl groups were effectively inserted on the CNT skeleton by a facile purification and modification. After ultrasonic dispersion, the modified pCNTs (M-pCNTs) were uniformly dispersed and loaded in the EP matrix. Better mechanical properties than EP were attained with a Young’s modulus of 3776.9 MPa, a tensile strength of 37.3 MPa, a fracture strain of 6.32%, and a fracture strength of 111.7 MPa with 2 wt % M-pCNT loading. Thus, pCNTs enhanced the toughness of the EP composites and simultaneously retained the stiffness. It was suggested that CNT pull-out and bridging were predominant toughening mechanisms for pCNT/EP composites. Notably, the coated film developed between residual metal in M-pCNTs and EP built a strong interfacial interaction and reinforced the EP composites.

## Abstract 2

Modified carbon nanotubes from waste plastic were applied as a filler to enhance the mechanical performance of epoxy resin composites.

## Introduction

Plastic pollution, the apparent tip of the iceberg, has become one of the most pressing environmental issues. In principle, the volumes of plastics discarded could be recaptured and reused, and the potential of plastic waste could be realized rather than landfilling or incinerating. Among the recycled technologies, converting waste plastics into high-value CNTs using chemical vapor deposition has been proved technically feasible.^1−3^ CNTs are becoming an important material in various high-end fields, including extreme sports, martial weapons, aerospace, and even space elevators, due to their light weight, robustness, and electrical and thermal conductivity while remaining chemically stable. In particular, due to the remarkable mechanical properties of CNTs (Young’s modulus ≥ 1 TPa and tensile strength ≥ 100 GPa), CNTs are one of the strongest known materials and the most ideal and promising reinforcements for improving the mechanical characteristics of resultant polymer composites.^4^

Epoxy resin (EP), possessing superior strength, adhesion, and insulation, is critical for airplanes, space shuttles, electronics, and other industrial uses. However, EP has limits, such as overfragility in applications; thus, additional components (e.g., glass, carbon, etc.) are needed to improve its performance.^5^ CNT/EP composites are preferable among reinforced epoxy systems because of their great strength, low weight, and versatility.^6^ Earlier studies have found that the mechanical properties of epoxy resins can be enhanced with small additions of carbon nanotubes (usually less than 10 wt %).^7,8^

Although the mechanical reinforcement is strongly reliant on the loading of carbon nanotubes, the degree of reinforcement may be limited at high loadings by the high viscosity of the polymer matrix and the subsequent void defects.^9^ In addition, the dispersivity of CNTs is a prerequisite to achieving sufficient dispersion. However, due to van der Waals interactions, CNT components tend to get entangled or overlay with each other, wasting the intrinsic strength of single CNTs.^10^ For example, several preparation methods, including solution mixing, failed to produce a sufficient dispersion of CNTs in the EP matrix.^11^ In recent years, sonication has been widely used to disperse CNTs and achieved better dispersion.^7^ It was found that the excellent mechanical properties of the composites primarily rely on interfacial interactions between CNTs and polymer matrices.^12^ By applying pretreatments, the dispersion of CNTs could be improved through strong interfacial adhesion with EP.^13^ Therefore, to minimize the agglomeration propensity and promote uniform distribution, the functionalization of CNTs has been thoroughly studied to modify CNTs before adding them to the polymer matrix.

The mechanical performance of the nanocomposite is closely connected to the polarity of CNTs.^14^ Due to the presence of oxygen groups on the surfaces, the polarity of CNTs can be enhanced by simple acid treatment. Hence, the surface polarity of carboxylated CNTs was superior to that of plain CNTs.^15^ In addition, carboxylation makes aggregate smaller, preventing the agglomeration tendency caused by the larger particle size.^12^ More importantly, the performance of the final product could be limited by the inherent quality of CNTs, e.g., purity, defect, and homogeneity. To date, many reported CNTs applied on epoxy resin composites showed diversity in mechanical results.^7,8^ The majority of epoxy resins employed high-quality CNTs prepared by catalytic decomposition of pure olefin gas like methane.^16^ However, few studies gave attention to CNTs prepared from waste plastic (pCNTs). The pCNTs have been proved to have a high yield and graphitization quality but a lower purity and inferior quality than commercial CNTs (cCNTs) due to the complexity of plastic polymers.^2,3,17^ Purification techniques like chemical oxidation have been widely used for obtaining desired purified CNTs, yet sufficient purification of pCNTs without damaging nanotubes is still a challenge.^1,3^ Thus, it is imperative to have an insight into the enhancement of raw pCNTs after basic pretreatment on final mechanics of EP composites, which may conversely give a direction for production and processing techniques of pCNTs.

This study researched carbon nanotubes produced from waste polypropylene, followed by purification and surface modification. Subsequently, the prepared CNTs were used to prepare CNT/EP composites to enhance the mechanical properties of the materials. Commercial CNTs, commercial modified CNTs (cMCNTs), and commercial carboxylated CNT/EPs (standard CNT/EP composites) have also been used as contrast groups. The properties of the modified pCNTs were characterized by several techniques, including TEM, TGA, XPS, and FTIR. The elastic characteristics of the pCNT/EP composites were then investigated in terms of mechanical measurement, including Young’s modulus, tensile strength, fracture strength, and fracture strain. Finally, the mechanism of mechanical enhancement was proposed by analyzing the morphology and surface physicochemical properties of the CNT/EP composites.

## Experimental Section

### Materials

The pCNTs were produced from waste polypropylene pyrolysis via the chemical vapor deposition (CVD) method in a two-stage fixed-bed reactor. The plastic samples were pyrolyzed first and the volatiles passed through a catalytic stage. In the presence of an Fe-based catalyst, the carbon sources were converted to CNTs. More details could be obtained in our previous work.^2^ Pristine cCNTs and cMCNTs (carboxyl content: 2.0 wt %) were purchased from Nanjing XFNANO Material Tech Co., Ltd. (Nanjing, China); both have 95% purity, a 10–30 μm length, and a 10–20 nm diameter. Commercial carboxylated CNT/EP was bought from Shanghai Aladdin Bio-Chem Technology Co., Ltd. (Shanghai, China). The chemicals used in this study include sulfuric acid (98% v/v, analytical grade) and nitric acid (67% v/v, analytical grade), which were analytical grade and ready for use. The epoxy resin E51, a low viscosity agent (EV184–195 g mol^–1^, viscosity 10 000–16 000 MPa s), and an amine curing agent (LEWEI 593, 500–600 mg of KOH g^–1^) used as the matrix were obtained from the Shanghai Autun Chemical Technology Co., Ltd. (Shanghai, China).

### Purification and Modification of Carbon Nanotubes

The pCNT samples consist of different sizes of aggregates with impurities. cCNTs (1 g) and crude pCNTs (1 g) were purified in a water bath, respectively, for 24 h at 40 °C with 150 mL of 20 wt % HNO_3_ to eliminate the undesired amorphous carbons and residue metallic catalysts.^7^ After the purification, the solution was vacuum filtered and rinsed with H_2_O, then finally dried under ambient pressure at 80 °C for 4 h. The samples were labeled as P-cCNTs and P-pCNTs. Mixed acids were used to chemically modify 0.5 g of purified cCNTs and pCNTs. The CNT samples were suspended in 80 mL of a 3:1 (v/v) mixture of concentrated H_2_SO_4_ (98 wt %) and HNO_3_ (67 wt %) and sonicated for 5 h at ambient temperature. After that, a filter membrane (0.2 μm pore size) was used to vacuum filter the mixture, which was then rinsed with deionized water until pH 7 and placed in the oven for 4 h at 80 °C before being used. These samples were labeled as M-cCNTs and M-pCNTs, respectively, corresponding to the purified cCNTs and pCNTs. Therefore, six samples were used in this work, including cCNTs, pCNTs, P-cCNTs, P-pCNTs, M-cCNTs, and M-pCNTs, representing commercial CNTs, produced CNTs, purified commercial CNTs, purified prepared CNTs, modified commercial CNTs, and modified prepared CNTs, respectively.

### Fabrication of Carbon Nanotubes Reinforced Epoxy Resin Composite

The CNT samples were manually blended in EP first, then sonicated for 4 h using an ultrasonic machine with high power (500 W, 40 kHz). At the end of the ultrasonication, a curing agent was added into the CNT/EP mixture at a weight ratio of 6:1 (EP/curing agent). After manually stirring for 5 min, the blend was transferred into a vacuum oven and degassed for 25 min. Then, the fluid CNT/EP composite was poured into an open stainless-steel mold. Thereafter, the viscous specimens were cured in an oven under air pressure with the temperature rising from 25 to 120 °C within 1 h and kept at 120 °C for 4 h. Figure S1 depicts the shape and size of the mold. Finally, the flat composite specimens shaped like a dog bone and rectangular flat composite samples were obtained. Figure S2 shows the schematic diagram of CNT treatment and composite fabrication. The tests were repeated three times to ensure the repeatability of results with less than 1% relative standard deviation.

### Characterization of CNTs

A transmission electron microscope (TEM) was used to observe the surface morphologies of cCNTs and pCNTs with a Tecnai G2 F20 S-TWIN instrument with a 200 kV accelerating voltage. Raman spectroscopy (Horiba Jobin Yvon, France) was conducted using a LabRAM HR 800 Evolution, equipped with an argon laser (532 nm) in the Raman spectra range of 200–3500 cm^–1^. Surface chemical analysis was performed with a Nicolet iS10 FTIR spectrometer with a 1:50 mg/mg KBr pellet and X-ray photoelectron spectroscopy (XPS, Axis Ultra DLD, Kratos). Thermogravimetric analysis (TGA) was carried out using a PerkinElmer Diamond TG/DTA Instrument with a heating rate of 10 °C min^–1^ in an air environment to determine the purity of the carbons.

### Characterization of CNT/EP Composites

An electronic universal material testing machine (Instron 5967, USA) was used to assess the mechanical characteristics of the CNT/EP composites. Both tensile and bending tests were performed at room temperature with a constant cross-head rate of 2 mm per minute. For each sample, three specimens were tested, and the average value was calculated using the data acquired. The fracture surface of CNT/EP composites was observed using a high-resolution scanning electron microscope (HRSEM, HitachiS4800, Japan) after sputter coating with an ion sputter coater (Hitachi Ion Sputter Coater E-1045, Japan).

## Results and Discussion

### Texture and Surface of CNTs

**Figure 1:**
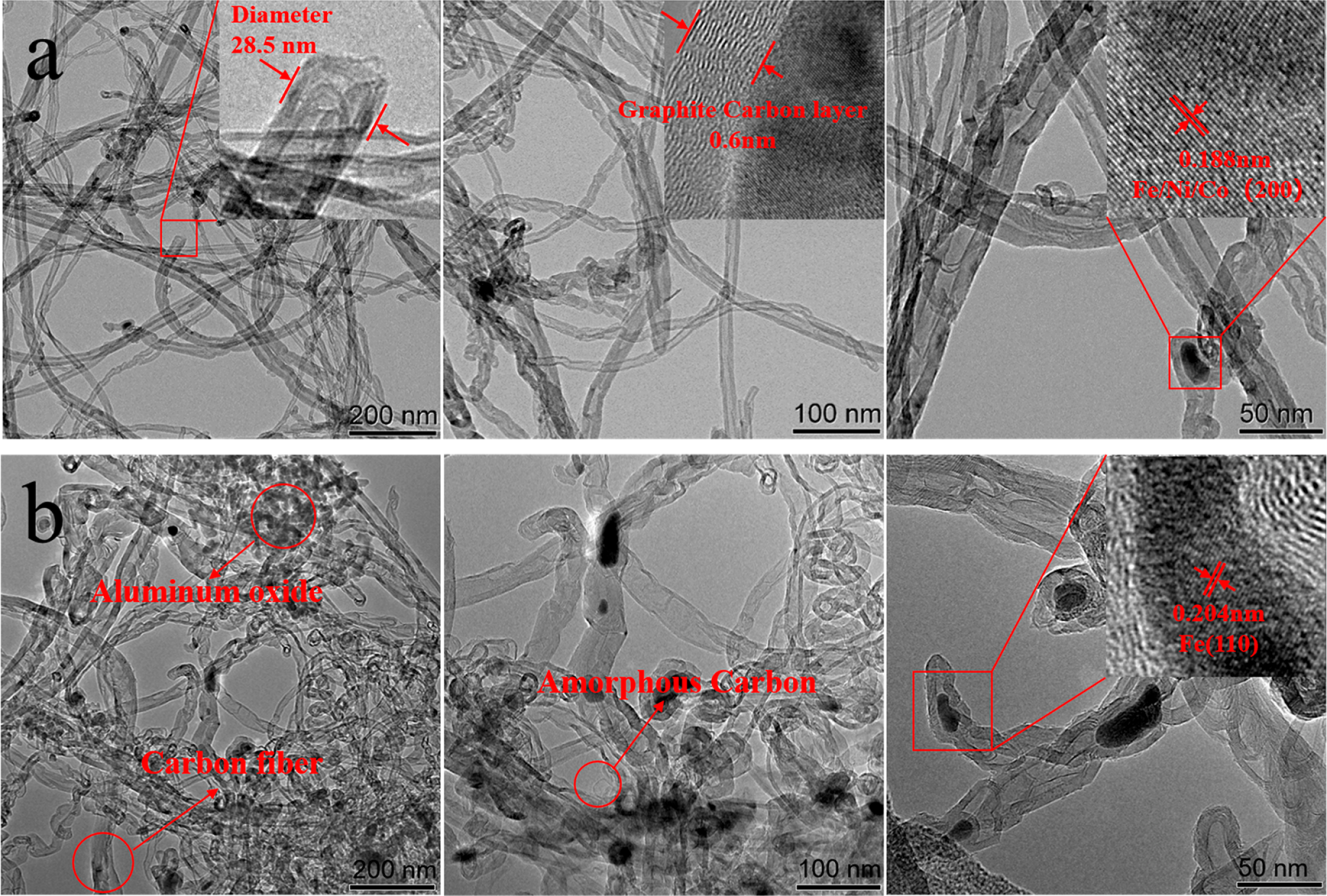
TEM of pristine (a) cCNTs and (b) pCNTs.

TEM images of the pristine cCNTs and pCNTs are shown in Figure 1. cCNTs (Figure 1a) show an excellent dispersion and clean CNTs with a small amount of ferronickel catalyst remaining inside the nanotubes.^18^ For the pCNTs, much more intertwined CNTs were observed in Figure 1b. The complexity of intermediate gaseous products from the conversion of waste plastic might be ascribed to the distortion and rough surface of the CNT tubes. As observed in the TEM results, the wrinkled structure of the pCNT surface could enhance the binding force between pCNTs and the matrix.^19^ In addition, from Figure 1b, carbon fibers and amorphous carbons were noticed from pCNTs. Notably, from the Figure 1b inset, residue aluminum oxide and iron remained in pCNTs, making it difficult to separate each other due to the strong affinity between aluminum oxide, iron, and the CNTs.^20^ In addition, as shown in Figure S3, the length of pCNTs is at least 20 μm, and the overall size and homogeneousness of pCNTs in Figure 1b were nearly identical to those of cCNTs in Figure 1a. Alternatively, pCNTs showed similar outer diameters ranging from a few nanometers to tens of nanometers and lengths reaching several micrometers at lower magnifications to cCNTs.

### Thermal Stability of CNTs

**Figure 2:**
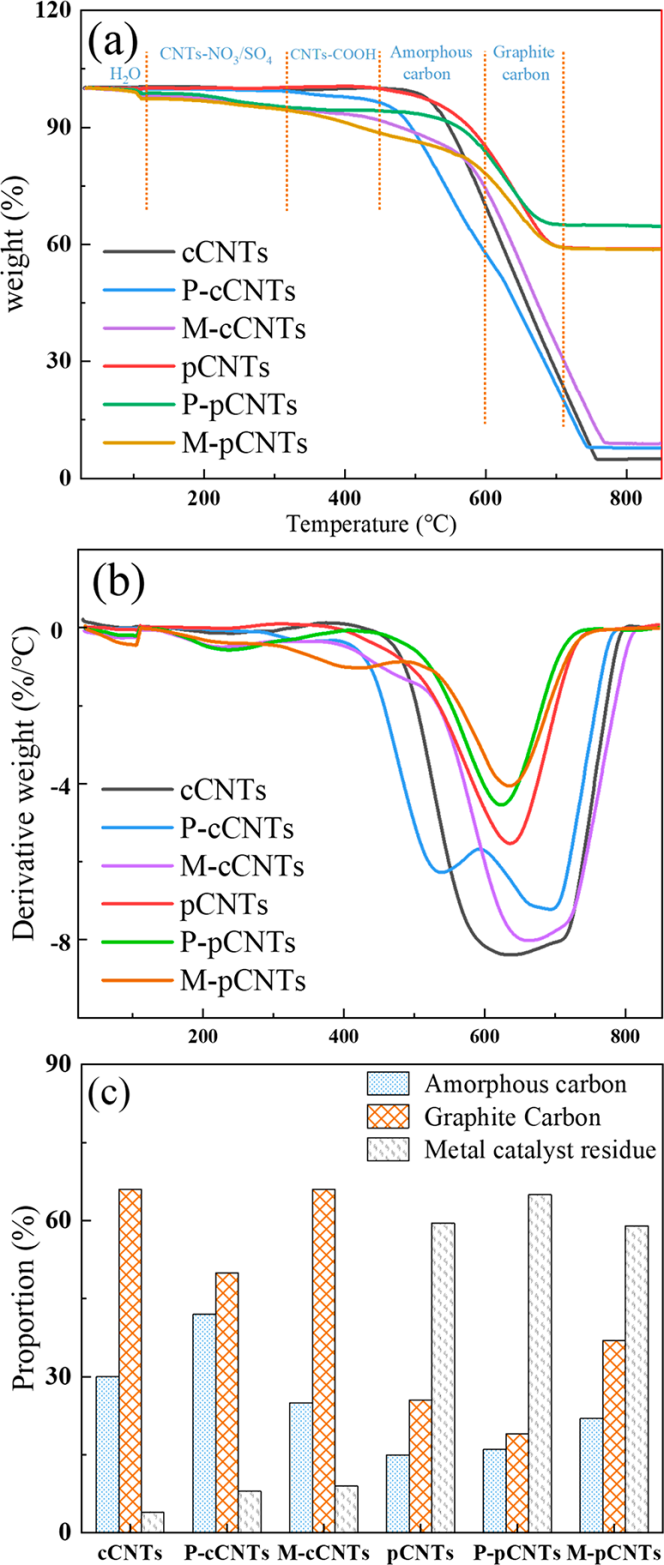
Thermal stability of cCNTs and pCNTs after different treatments. (a) TGA and (b) possible proportions of compositions.

Figure 2 illustrates the composition of CNTs after the modification processes. The TGA and DTG curves of all samples are shown in Figure 2a and b, respectively. When the temperature was less than 110 °C, no apparent weight loss occurred except for an initial slight weight loss due to water evaporation. Then M-cCNTs, P-pCNTs, and M-pCNTs started degrading at around 110 °C, whereas for the other samples, this value was 320 °C. According to the TGA thermograms of cMCNTs in Figure S4, the weight loss of cMCNTs started from 320 °C, attributed to the loss of other oxygen-contained groups like COOH in the CNT skeleton. After that, all samples showed significant weight loss with the increase of temperature. There was a noticeable drop in mass with increasing temperature (475–600 °C), particularly for the deposited carbon that was unstable amorphous carbons. With the further increase of temperature (>600 °C), graphite oxidation was responsible for the weight reduction. However, pCNTs contained abundant stable metal catalyst materials, including aluminum support and ferric oxide, resulting in less mass reduction than cCNTs.^2^ Also, it was seen that at the final temperature (775 °C), cCNTs, P-cCNTs, and M-cCNTs degraded to below 10% of the initial value, whereas for pCNTs, P-pCNTs, and M-pCNTs, the corresponding values were 40.5%, 35%, and 41%, respectively.

As shown in Figure 2a and b, the weight loss of the various samples corresponded to the content of the residual metal catalysts in Figure 2c. In addition, for cCNTs, the results of the derivative weight of P-cCNTs showed two main peaks around 600 °C. The first peak was assigned to the combustion of the amorphous carbons. cCNTs and M-cCNTs reached the lowest weight-loss ratio from 600 °C and retarded around 635 °C, which could be associated with the oxidation of amorphous carbons coated on graphite carbons.^21^ Then, when the TGA temperature was further increased, graphite carbons began to burn and lose weight. As shown in Figure 2c, the commercial CNTs (cCNTs, P-cCNTs, M-cCNTs) had a higher proportion of graphite carbons (over 50–75%), whereas the prepared CNTs (pCNTs, P-pCNTs, M-pCNTs) had a lower proportion of graphite carbons (about 20–40%). Simultaneously, approximately 20% of amorphous carbons and 60% of metal catalyst residues were found in pCNTs. It is noted that high metal content in pCNTs existed after the purification and carboxylation. It is suggested that the purification removed Fe outside of the carbon nanotubes, whereas Fe inside nanotubes still existed. Meanwhile, the remaining metal in M-pCNTs was aluminum oxide, which is hard to efficiently remove in the purification process.

### Skeleton Structure of CNTs

**Figure 3:**
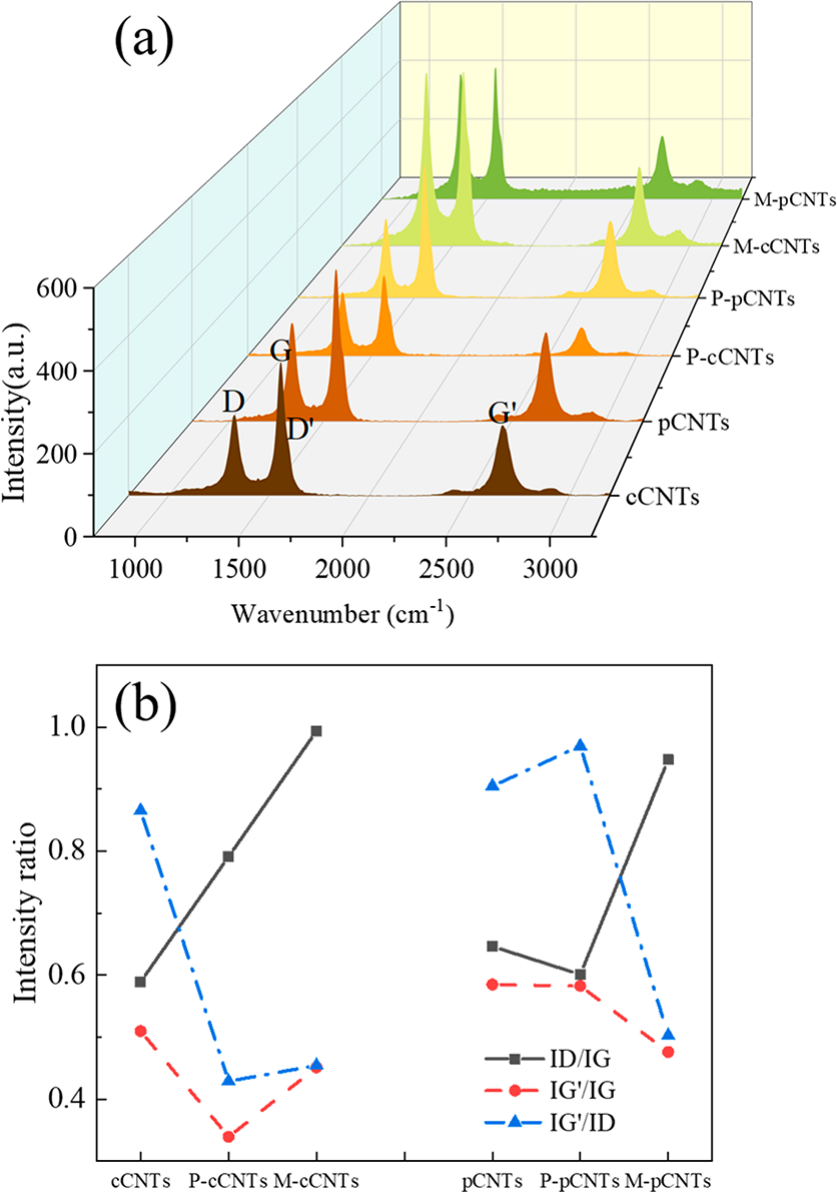
Raman of (a) cCNTs and pCNTs after different treatment and (b) relative intensity ratios between each band.

Figure 3 shows Raman spectra analysis of cCNTs and pCNTs under different conditions. All Raman spectra have shown four bands, including the D band (1350 cm^–1^) and G band (1580 cm^–1^), the D′ band (1620 cm^–1^) near a shoulder at the G band, and the G′ band (2700 cm^–1^). The D band reflects the defects and noncrystalline carbon; the stretching mode of sp^2^ carbon atoms of CNTs matches the G band. As shown in Figure 3a, modified CNTs showed higher D and G band intensities, reflecting an increase in the proportion of CNTs and defect sites. The sp^3^ hybridized carbon without in-plane symmetry connected with the D′ band, and the G′ peak was considered a unique signal linked to sp^2^-carbon bonds.^19,22^ It is noted that the presence of graphene on the CNTs may explain the high G′ band.^19^ There was no apparent change of D′ and G′ for different CNTs in Figure 3a, demonstrating a barely visible change of graphene in the CNT samples.

Figure 3b shows the relative intensity of the D and G bands (*I*_D_/*I*_G_), G′ and G bands (*I*_G′_/*I*_G_), as well as G′ and G bands (*I*_G′_/*I*_D_) ratios. The CNTs samples were evaluated by comparing *I*_D_/*I*_G_, which was related to the number of structural defects and sp^3^ hybridized carbon atoms in a CNT sample. This ratio provided a clear picture of the functionality degree of the sidewalls of graphitic materials. Therefore, a rise in *I*_D_/*I*_G_ indicated a reduction of aromaticity (oxidation of sp^2^ hybridized carbon) in CNT rings. The change in the hybridization of sp^2^ for sp^3^ C–C due to the presence of oxygen or other atoms in CNT structure caused the defects of materials and might lead to the loss of aromaticity. The increase of the *I*_D_/*I*_G_ ratio of the modified CNTs compared with that of the pristine CNTs confirmed the successful introduction of functional groups onto the CNT surface and that the outer layers of the CNTs were chemically modified. Moreover, *I*_G′_ /*I*_G_ and *I*_G′_ /*I*_D_ were linked to the graphitic order scale and crystallinity, respectively. There were more lattice defects in the functionalized CNTs, as evidenced by the lower ratios of *I*_G′_ /*I*_G_ and *I*_G′_ /*I*_D_.^23^

### Chemical Bonding Structure of CNTs

**Figure 4:**
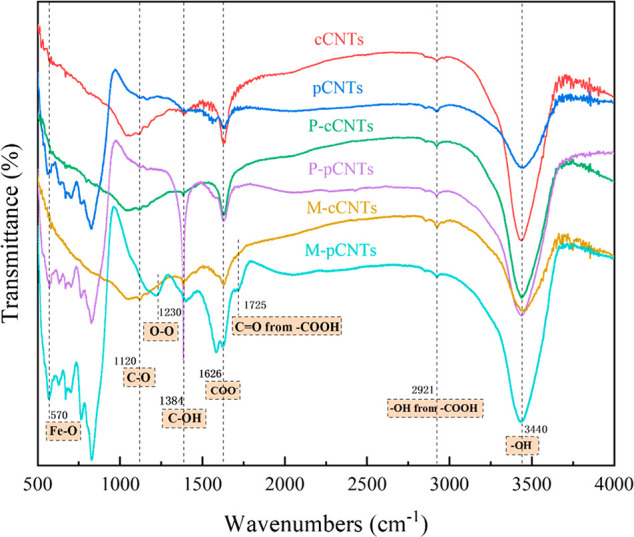
FTIR of cCNTs and pCNTs after different treatment.

Further information regarding the chemical bonding structure was obtained from FTIR spectroscopy. Figure 4 shows the FTIR spectra of cCNTs and pCNTs. Compared to the functionalized CNTs, raw CNTs showed few IR signals. A band around 1120 cm^–1^ was attributed to the C=C bond in the skeletal vibration mode of CNT. Then, a weaker feature at 1230 cm^–1^ was assigned to C–O stretching and O–H bending vibrations (CNT–COOH). Notably, another principle band, 1626 cm^–1^, was related to the carboxylate anion stretch mode (−COO^–^; possibly attributed to the skeletal CNT interaction with carboxyl and ketone groups), while 1725 and 2921 cm^–1^ were assigned to the C=O stretch mode and the O–H stretch of −COOH, respectively.^24^ The presence of the carboxyl group on the surface of cCNTs could be due to the partial oxidation during purification, whereas the pristine pCNTs showed the presence of a trace carboxyl group, which could be a result of the pyrolysis of waste plastic. After carboxylation, the subtle intensity of these peaks increased significantly, indicating oxidation reactions on the nanotube surfaces. In addition, the peak attributed to the C=C bond maintained at 570 cm^–1^ showed a CNT–COO–FeO bond.^24^ This Fe–O peak revealed that part of the catalytic metallic nanoparticles inside the hollow CNTs was possibly eliminated because the purification process removed the nanotube caps. Then, Fe coexisted with CNTs through bridging interactions between carboxyl groups and Fe. In addition, in all CNTs, a high absorption band appeared at ∼3440 cm^–1^, which was a characteristic of the O–H stretch of the hydroxyl group.^25^ In addition, the bending of hydroxyl groups was also found in the peak of 1384 cm^–1^.^26^ It is noted that the O–H group could also be affected by the adsorbed water of CNTs.^27^

**Figure 5:**
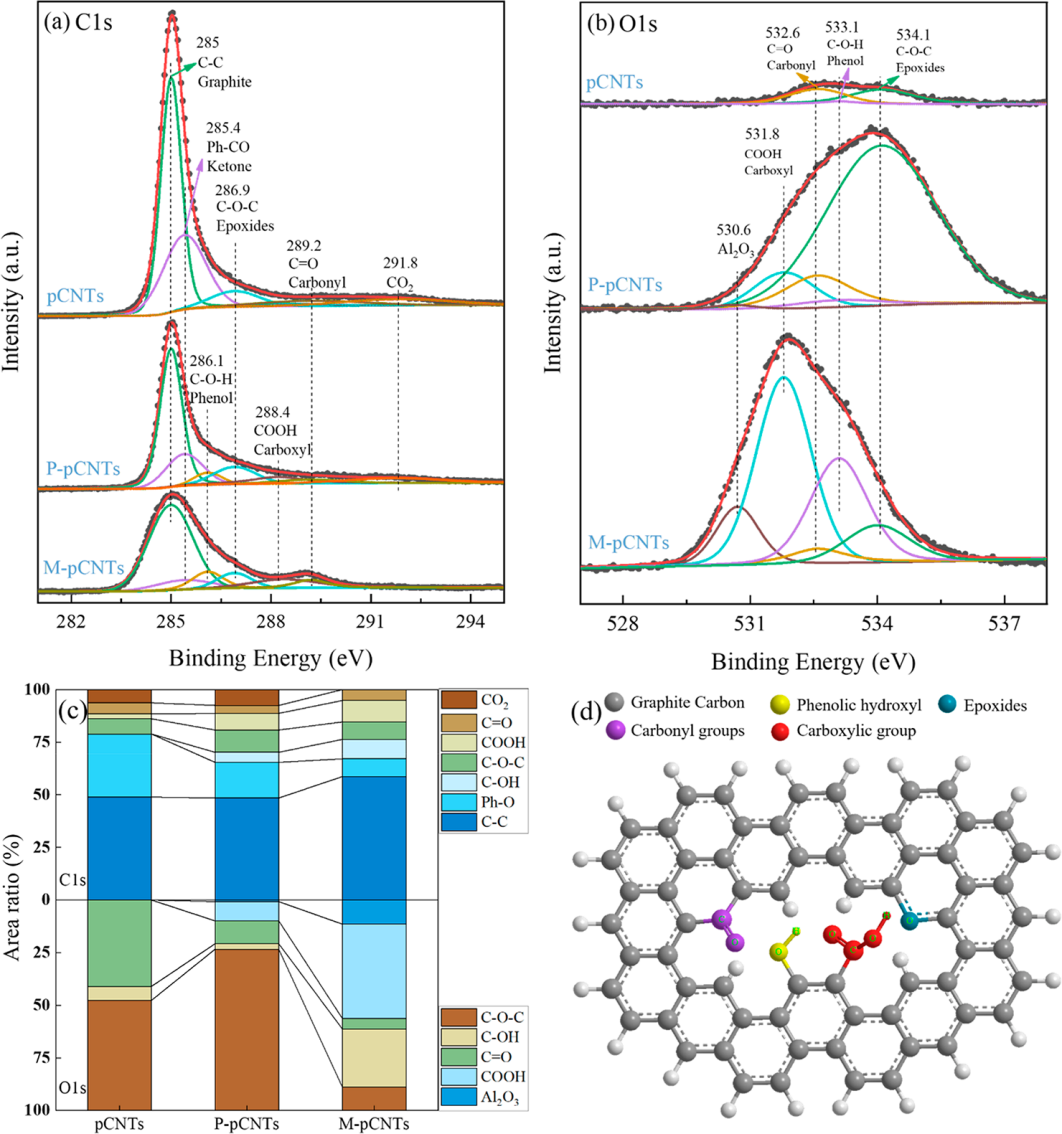
XPS result of pCNTs (a) C 1s, (b) O 1s, and (c) main groups area ratios of pCNT, P-pCNT, and M-pCNT spectra. (d) M-pCNT structure containing possible functional groups.

XPS analysis was carried out to understand the surface chemicals on the CNT samples. According to the XPS figure of C 1s, O 1s, N 1s, and S 1s spectra of CNTs in Figure S5, CNTs were mainly composed of C and O elements. The elemental composition, chemical state, and electronic state of CNTs were also investigated. The C 1s and O 1s spectral deconvolution spectrum of pCNTs were recorded in Figure 5a and b, respectively. For the original samples without pretreatment, C 1s spectra were dominated by conjugated C–C bonds (285 eV), which decreased obviously after the purification and modification. These spectra were attributed to graphite carbons and amorphous carbons, which were also identified based on the aforementioned TGA results in Figure 2.^28^ In addition, the intensity of Ph–C=O at 285.4 eV reduced along with conjugated C–C bonds, implying that the ketone group arising from the pyrolysis gas product of waste plastic existed mainly on the surface of the sp^2^ carbon. The component at 286.9 eV was attributed to the epoxide group (C–O–C), showing little change. However, the components related to phenol (C–O–H, 286.1 eV) and carbonyl (C=O, 289.2 eV) were significant in M-pCNTs.^29^ More importantly, the intensity around 288.4 eV increased apparently in the purified CNTs and carboxylated CNTs^30^ compared to the untreated CNTs, further demonstrating that the carbon nanotubes were chemically modified with introduced functional groups. There was no other obvious difference among the C 1s spectrum for the studied CNT samples.

O 1s peaks tend to be broad with multiple overlapping components compared with C 1s peaks. The inherent existence of trace amounts of O elements in the pristine pCNTs might result from the preparation of CNTs. In addition, the hump shape of the O 1s spectrum suggested the existence of surface species in the purified and modified CNTs, possibly arising from the absorbed anions NO_3_^–^ and SO_4_^2–^. The first band at 530.6 eV might represent under-coordinated lattice O bound to Al and Fe, which was distinguished from other features after modification.^31^ Carbonyl (C=O, 532.6) was the main bond of pristine pCNTs, which remained nearly unchanged. Whereas two superior bands were ascribed to incompletely oxidized carbon, epoxides (C–O–C, 531.4) and phenol (C–O–H, 532.6), the intensity of them decreased and increased through the shearing of CNTs by a carboxylation step.^19^ Notably, the O 1s XPS spectrum of M-pCNTs presented an enhanced intensity of the carboxyl group (COOH, 531.8) after being treated by sulfuric and nitric acid, which was higher than that of pCNTs, illustrating that the introduced O results from the carboxyl group.^19^ Those changes indicated that the residual Fe catalysts were converted into oxides while the oxygen-containing functional groups on pCNTs were further oxidized by the carboxylation step.

Figure 5c represents the area ratio of main groups in the pCNT skeleton based on each fitted deconvolution spectrum. The proportion of the COOH group obviously increased from 2.3% to 10.2%. Also, COOH appeared in the O 1s deconvolution spectrum of P-pCNTs, then reached the maximum in M-pCNTs (44.8%). Compared with pristine pCNTs, M-pCNTs showed the existence of the various oxygen-containing functional groups (Figure 5c and d). These oxygen-rich functional groups could provide a chemically reactive graphitic network on CNTs.^19^ The XPS results were consistent with the above FTIR results and confirmed the introduction of −COOH in CNTs.^25^

### Effect of CNTs Processing on EP Composites

**Figure 6:**
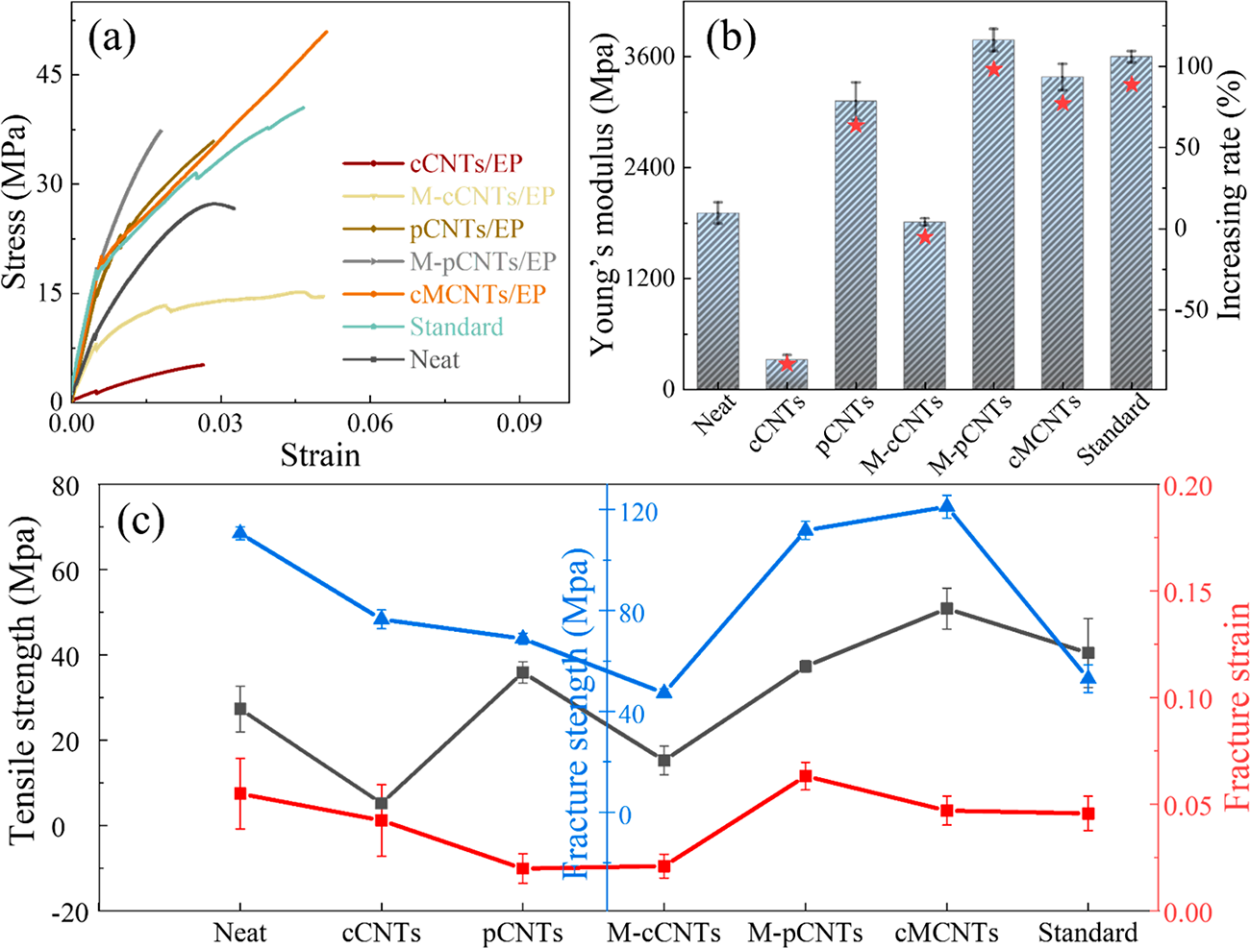
Mechanical results of CNT/EP composite added with 2 wt % cCNTs and pCNTs under different conditions. (a) Representative strain–stress curves. (b) Young’s modulus. (c) Tensile strength, fracture strength, and fracture strain.

In this study, the mechanical properties of CNT composites depended on the dispersion and interfacial interaction between CNTs and epoxy resin. Tensile and flexural tests were carried out to obtain the mechanical properties of EP composites. The strain–stress curves were acquired after a break of the EP composite occurred. Figure 6a shows the representative strain–stress curves of neat EP and CNT/EP composites with 2 wt % different CNTs added. It is demonstrated that all the strain–stress curves exhibited a linear elastic behavior and a plastic deformation. The largest area under the strain stress curve of the commercial modified CNT/EP composites indicated excellent toughness. The steepest strain stress curve in Figure 6a showed that M-pCNT/EP is strongest than other CNT/EP composites. Compared with cCNTs, pCNTs imparted superior stiffness but inferior ductility to EP. There was a lower actual content of carbon nanotubes in pCNTs than in cCNTs, which resulted in good dispersion of pCNTs in EP. Conversely, high content caused poor dispersion of CNTs in EP, impairing the original mechanical properties of CNTs.

As shown in Figure 6b, the pCNT/EP composites had the highest value of Young’s modulus among EP composites. In addition, compared with cCNT/EP, pCNT/EP showed better performances in relation to Young’s modulus, tensile strength, fracture strain, as well as fracture strength, implying that pCNTs were more tenacious and flexible (at the same 2% concentration; Figure 6b,c). In addition, after the modification, M-pCNT/EP showed better mechanical performance compared with M-cCNT/EP. However, the poor mobility of M-cCNT/EP at a high loading content caused difficulty for sample preparation (casting). And more defects were introduced during the preparation of M-cCNT/EP composite specimens, which partly explained its worse mechanical performance than M-pCNT/EP. Particularly, M-pCNT/EP showed a higher Young’s modulus and fracture strain but lower tensile strength and fracture strength compared with cMCNT/EP, which can be attributed to the carboxylation of residue metals in pCNTs during the modification. Carboxylation of pCNTs might cause acid etching on residue metal catalyst, forming a strongly adhered film on the metal surface through its multi-coordination sites, then the adhesive force between metal and the epoxy resin matrix was enhanced.^32,33^ Meanwhile, the coated metal and CNT agglomeration made the M-pCNT/EP composite more inhomogeneous and easier to break than the cMCNT/EP composite. Notably, the mechanical performance of M-pCNT/EP slightly outweighed the standard commercial carboxylated CNT/EP composite.

### Effect of CNTs Loading Content on EP Composite

**Figure 7:**
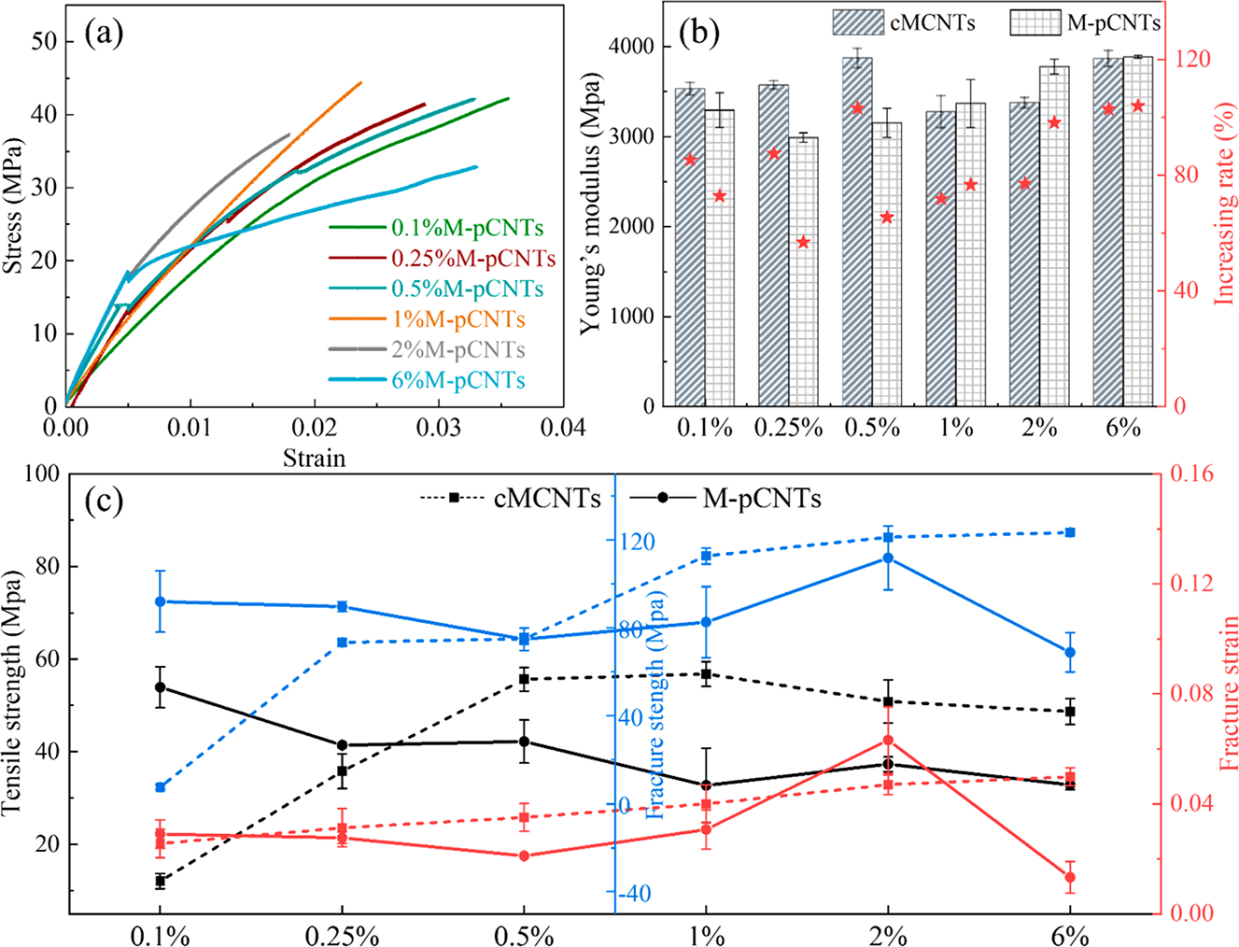
Mechanical characterization of CNT/EP composite with the addition of CNTs at different ratios. (a) Representative tensile strain–stress curves. (b) Young’s modulus. (c) Tensile strength, fracture strength, and fracture strain.

In this section, the influence of M-pCNT loading on the mechanical performances of epoxy composites was studied. Table S1 presents the detailed proportion gradient of M-pCNTs and EP in the CNT/EP composites. Figure 7a shows a strain–stress curve of composites with different loadings of M-pCNTs. The curve became steeper when more M-pCNTs were added and leveled off at 6 wt %. All of the M-pCNT/EP composites showed gentle stress–strain curves and a rise in Young’s modulus compared to the neat epoxy. In other words, the modified pCNTs underwent an efficient shear-loading through carboxylation, resulting in an increased tenacity.

In Figure 7b, the value of Young’s modulus of the M-pCNT/EP composite tended to rise with the addition of M-pCNTs from 0.1 wt % to 6 wt %. The stiffness of EP was strengthened by M-pCNTs, evidenced by Young’s modulus increasing from 1906.5 to 3887.3 MPa. In contrast, Young’s modulus of the cMCNT/EP composite rose slowly when cMCNTs’ loading increased from 0.25 wt % to 0.5 wt %. As seen from Figure 7c, although the highest stiffness of cMCNT/EP was obtained at a 0.5 wt % loading of cMCNTs, the maximum improvement of tensile strength, fracture strength, and fracture strain were found at 1–2 wt % cMCNTs loading. When the cMCNT level increased from 0.5 wt % to 1 wt %, the amount and size of cMCNTs aggregates in the cMCNT/EP composite might increase, which reduced the interfacial bonding and the wettability between cMCNTs and the EP matrix, resulting in the reduction of the stiffness of the cMCNT/EP composite. While the cMCNT loading was between 1 wt % to 2 wt %, it is suggested that more cMCNTs would enhance the mechanical performance. This could be attributed to the higher content of carbon nanotubes in cMCNTs than pCNTs when the same weight ratio of CNTs was added to the composites. The M-pCNT/EP composite with a lower CNT loading content (at 0.1 wt %) showed a relatively higher tensile strength (53.9 MPa) than other loading contents. However, the fracture strength and fracture strain of the M-pCNT/EP composite experienced a moderate fluctuation, then peaked at a higher content level (2 wt %); the value of fracture strength and fracture strain was 111.7 MPa and 63.17%, respectively. Meanwhile, the M-pCNT/EP composite at 2 wt % loading content of M-pCNTs showed a relatively high Young’s modulus and tensile strength. When the loading of M-pCNTs was higher than 2 wt %, both values of the tensile and flexure performance of the M-pCNT/EP composite began to decrease.

### Interface Interaction of M-pCNT/EP Composites

**Figure 8:**
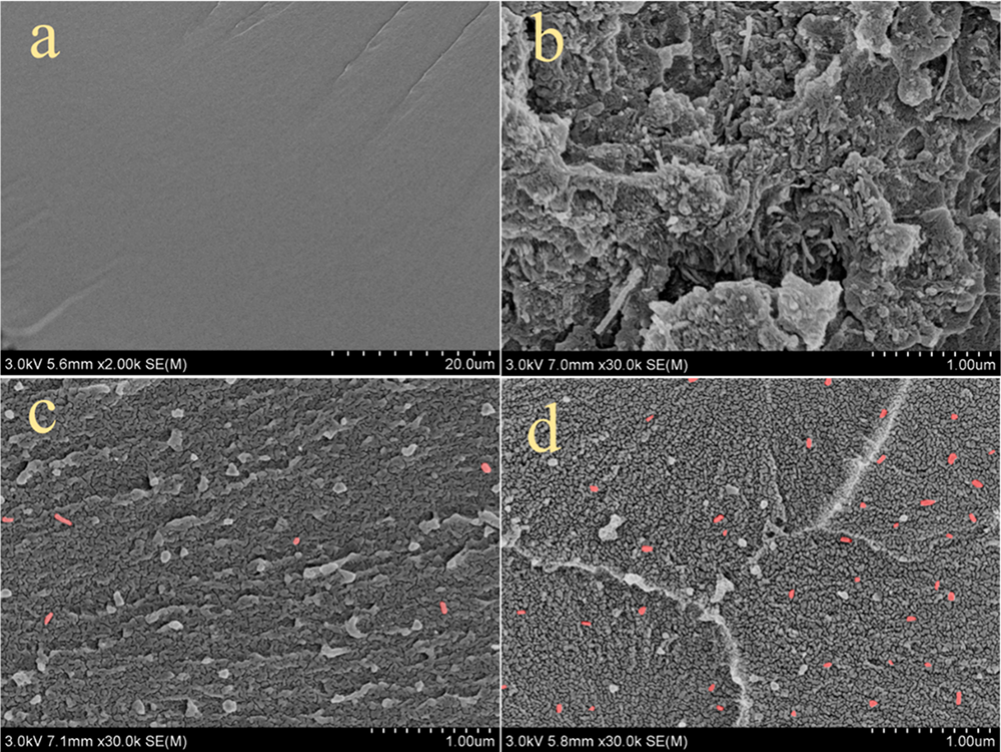
SEM images of fracture surface of (a) neat epoxy resin, (b) the M-pCNTs agglomeration in EP matrix, (c) 0.1 wt % M-pCNT/EP composite, (d) 6 wt % M-pCNT/EP composite.

Figure 8 shows the morphologies of the studied M-pCNT/EP composites. Apparently, unlike the smooth surface of the neat epoxy resin (Figure 8a), CNTs introduced a rough surface to the EP composites (Figure 8c and d). In general, the rough fracture surface indicated strong tensile strength, while the smooth surface typically was related to low tensile strength. In addition, M-pCNTs were agglomerated within EP, as shown in Figure 8b. However, M-pCNTs were separated into individual nanotubes (marked with the red shadow) due to the shearing force related to carboxylation. Thus, M-pCNTs distributed homogeneously and became abundant when the content of M-pCNTs in the composite increased from 0.1 wt % to 6 wt %. Furthermore, the river-like texture of the 2 wt % M-pCNT/EP composite indicated the tendency of plastic deformation (Figure 8d). It was demonstrated that a higher content of M-pCNTs (∼6 wt %) could make the composites more flexible, which was close to the standard cMCNT/EP composite.

**Figure 9:**
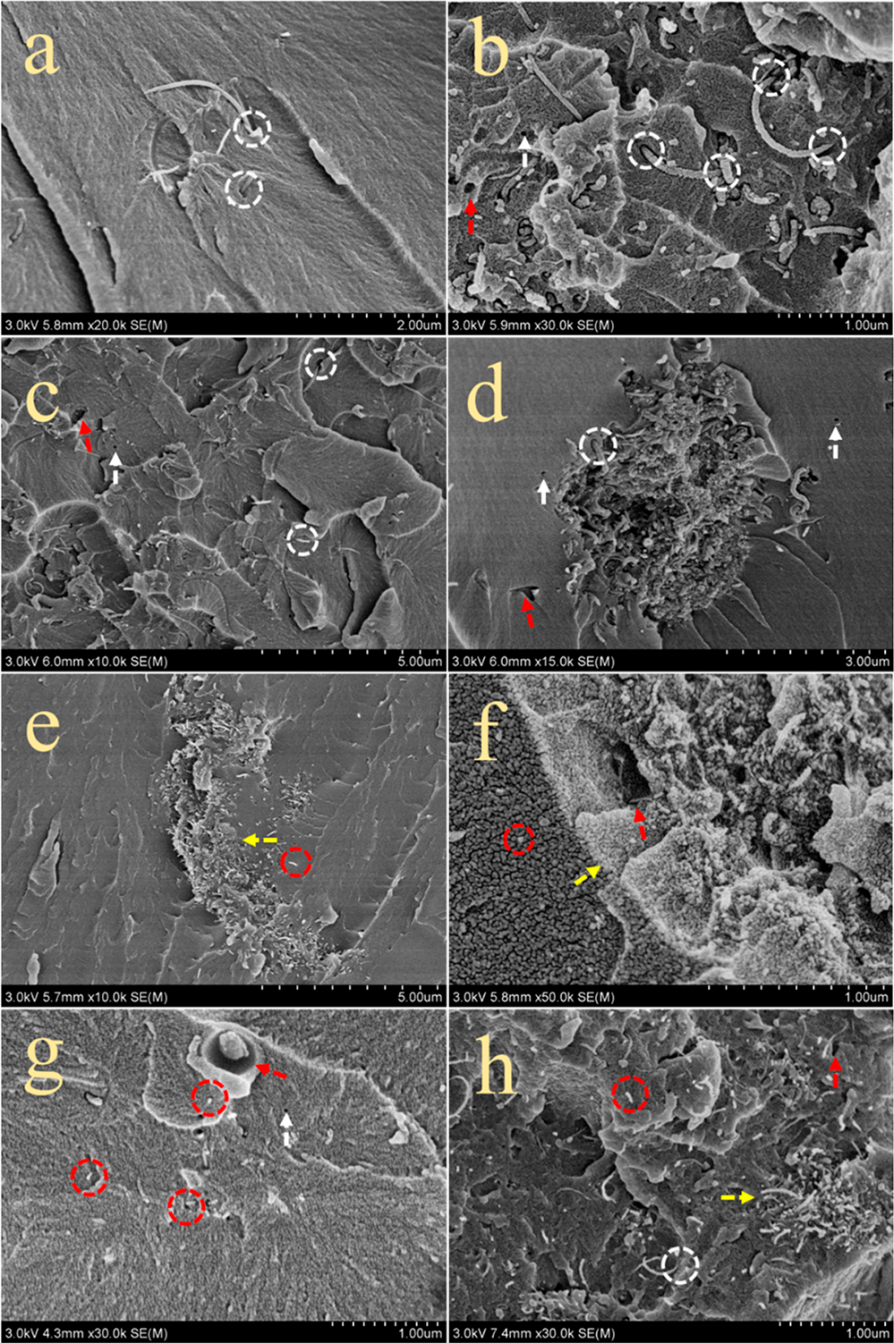
Fracture surface of epoxy composite with (a) cCNTs, (b) pCNTs, (c) purified cCNTs, (d) purified pCNTs, (e) functionalized cCNTs, (f) functionalized pCNTs added composites, (g) cMCNTs, and (h) standard commercial CNTs carboxylated EP.

In Figure 9a–d, cCNTs and pCNTs, circled in white, were long and curled with two sides embedded in the epoxy matrix, which can be explained as CNT bridging. More CNT bridging was observed in the pCNT/EP composites than the cCNT/EP composites, corresponding to better stiffness and inadequate fracture performance of the pCNT/EP composites in Figure 6. As illustrated by the white arrows in Figure 9, some CNTs peeled away from the matrix during CNT loading, creating small holes in the matrix, especially in the vicinity of the aggregates. Red arrows showed widespread defects resulting from bubbles and aggregates. It can be explained that a high loading of CNTs caused insufficient fluency and high viscosity of EP composites and poor interfacial connectivity in the region of the aggregates. After the purification and carboxylation, the well sheared and dispersed CNTs cause the CNT pull-out to increase and defects of CNT/EP composites to decrease significantly (Figure 9c–h). Hence, CNT modification imparted better mechanical properties to both cCNT/EP composites and pCNT/EP composites by the CNT bridging and CNT pull-out.^8^

Notably, as shown in Figure 9c,d, unlike pristine CNTs, the P-pCNTs have agglomerated with a distinctive boundary, and residual P-pCNTs were distributed individually in EP. This phenomenon could be caused by the different affinity between the metal catalyst and CNTs with epoxy resin. More metal residue in pCNTs than cCNTs contributed to the formation of an agglomeration boundary.^33^ Furthermore, after the carboxylation, the boundary (marked with yellow arrows) between pCNTs and EP became clearer with the observation of long and curled CNTs (Figure 9e,f). The CNTs became shorter due to the shearing action during the pretreatment of carboxylation, which alleviated the agglomeration of remaining pCNTs. At the same time, the boundary between CNTs and EP in the cCNT/EP composites and the standard cMCNT/EP composites could hardly be observed (Figure 9g,h).

Interfacial interaction at the CNT/EP composite was enhanced through the modification. Regarding the M-pCNT/EP composite, there was a strong interfacial bonding between the carboxylated carbon nanotubes and EP because of a strong p–p interaction of the carboxyl group.^34^ Meanwhile, EP inherently contained pendant hydroxyl groups alongside the molecular chain. These −OH species could form strong bonds or polar attractions to oxide or hydroxyl on the surfaces of metals. Additionally, the polarity of EP resulted in high surface energy. As a result, EP could permeate the incompact metal catalyst as an adhesive, then the holes on the surface of the M-pCNT agglomerate were filled. Naturally, the outer EP molecular across the whole surface of the boundary had a great cohesive force with the EP matrix due to mutual attraction. Hence, the formed adhered coated films contributed to the metal and EP boundry.^32,33^ In addition, any free hydroxyl groups on the surface of the Al_2_O_3_ nanoparticles (used for pCNTs preparation) could form hydrogen bonds with the oxygen in EP,^35^ which further validate the enhancement of mechanical properties in Figure 6. As a result, it is proposed that the remaining metal catalyst might enhance the interfacial interaction between CNTs and epoxy resin. Therefore, the agglomeration of M-pCNTs could be conducive to the tensile strength and fracture strength compared with M-cCNTs.

## Conclusions

This study investigated the difference between pCNTs and cCNTs and the efficiency of purification and modification on improving the mechanical properties of EP composites. An approximate 60 wt % of metal catalyst had remained in M-pCNTs, which suggested that the actual content of carbon in pCNTs was substantially lower than in cCNTs. As a result, pCNTs were easier to disperse and achieved better mechanical performance than cCNTs when the CNT loading was at 2 wt %. After purification and carboxylation, both cCNTs and pCNTs were shortened, and the carboxyl groups were successfully introduced to the carbon nanotube skeleton. Adding an appropriate amount of M-pCNTs was favorable to increasin Yong’s modulus, fracture strength, and fracture strain of neat EP. For example, after loading 2 wt % pCNTs, the modified pCNTs achieved the optimal mechanical properties with a Young’s modulus of 3776.9 MPa, tensile strength of 37.3 MPa, fracture strain of 6.32%, and fracture strength of 111.7 MPa. The optimal M-pCNTs loading content (2 wt %) was suggested for better mechanical properties. M-pCNTs achieved a better toughness than M-cCNTs as additives to EP composites at the same weight loading. Although M-pCNTs had a better stiffness, there was no big difference between M-pCNTs and cMCNTs on toughness affected by residue metal in pCNTs. It is further suggested that CNT pull-out and bridging were predominant toughening and reinforcement mechanisms for cCNT/EP and pCNT/EP composites. Notably, the residue metal in M-pCNTs strengthened interfacial bonding force due to the formation of coated film between metal in pCNTs and EP. Therefore, CNTs produced from waste plastics are equipped with outstanding mechanical properties comparable to commercial carbon nanotubes, which have great potential for mechanical applications.

## Supporting Information Available

The size of specimens for tensile and flexural test (Figure S1), schematic diagram of CNT treatment and composite fabrication (Figure S2), the length characterization of pCNTs (Figure S3), TGA thermograms of cMCNTs (Figure S4), XPS figure of elements spectra of different CNTs (Figure S5), and the composition of different CNT/EP samples (Table S1) (PDF)

## References

[ref1] Gou X.; Zhao D.; Wu C. Catalytic conversion of hard plastics to valuable carbon nanotubes. Journal of Analytical and Applied Pyrolysis 2020, 145, 104748 10.1016/j.jaap.2019.104748.

[ref2] Cai N.; Li X.; Xia S.; Sun L.; Hu J.; Bartocci P.; Fantozzi F.; Williams P. T.; Yang H.; Chen H. Pyrolysis-catalysis of different waste plastics over Fe/Al2O3 catalyst: High-value hydrogen, liquid fuels, carbon nanotubes and possible reaction mechanisms. Energy Conversion and Management 2021, 229, 113794 10.1016/j.enconman.2020.113794.

[ref3] He S.; Xu Y.; Zhang Y.; Bell S.; Wu C. Waste plastics recycling for producing high-value carbon nanotubes: Investigation of the influence of Manganese content in Fe-based catalysts. Journal of Hazardous Materials 2021, 402, 123726 10.1016/j.jhazmat.2020.123726. 33254760

[ref4] Arash B.; Wang Q.; Varadan V. K. Mechanical properties of carbon nanotube/polymer composites. Sci. Rep. 2015, 4 (1), 6479 10.1038/srep06479. PMC4180807 25270167

[ref5] Li L.; Zou H.; Shao L.; Wang G.; Chen J. Study on mechanical property of epoxy composite filled with nano-sized calcium carbonate particles. J. Mater. Sci. 2005, 40 (5), 1297–1299. 10.1007/s10853-005-6956-7.

[ref6] Zhang S.; Ma Y.; Suresh L.; Hao A.; Bick M.; Tan S. C.; Chen J. Carbon Nanotube Reinforced Strong Carbon Matrix Composites. ACS Nano 2020, 14 (8), 9282–9319. 10.1021/acsnano.0c03268. 32790347

[ref7] Guo P.; Chen X.; Gao X.; Song H.; Shen H. Fabrication and mechanical properties of well-dispersed multiwalled carbon nanotubes/epoxy composites. Compos. Sci. Technol. 2007, 67 (15), 3331–3337. 10.1016/j.compscitech.2007.03.026.

[ref8] Esmaeili A.; Ma D.; Manes A.; Oggioni T.; Jiménez-Suárez A.; Ureña A.; Hamouda A. M. S.; Sbarufatti C. An experimental and numerical investigation of highly strong and tough epoxy based nanocomposite by addition of MWCNTs: Tensile and mode I fracture tests. Composite Structures 2020, 252, 112692 10.1016/j.compstruct.2020.112692.

[ref9] Ma P.-C.; Siddiqui N. A.; Marom G.; Kim J.-K. Dispersion and functionalization of carbon nanotubes for polymer-based nanocomposites: A review. Composites Part A: Applied Science and Manufacturing 2010, 41 (10), 1345–1367. 10.1016/j.compositesa.2010.07.003.

[ref10] Bai Y.; Zhang R.; Ye X.; Zhu Z.; Xie H.; Shen B.; Cai D.; Liu B.; Zhang C.; Jia Z.; et al. Carbon nanotube bundles with tensile strength over 80 GPa. Nat. Nanotechnol. 2018, 13 (7), 589–595. 10.1038/s41565-018-0141-z. 29760522

[ref11] Allaoui A.; Bai S.; Cheng H. M.; Bai J. B. Mechanical and electrical properties of a MWNT/epoxy composite. Compos. Sci. Technol. 2002, 62 (15), 1993–1998. 10.1016/S0266-3538(02)00129-X.

[ref12] Roy S.; Petrova R. S.; Mitra S. Effect of carbon nanotube (CNT) functionalization in epoxy-CNT composites. Nanotechnol. Rev. 2018, 7 (6), 475–485. 10.1515/ntrev-2018-0068. 30637182 PMC6326190

[ref13] Liu X.; Wei B.; Farha F. I.; Liu W.; Li W.; Qiu Y.; Xu F. Densely packed, highly strain sensitive carbon nanotube composites with sufficient polymer penetration. Composites Part A: Applied Science and Manufacturing 2020, 130, 105728 10.1016/j.compositesa.2019.105728.

[ref14] de Menezes B. R. C.; Ferreira F. V.; Silva B. C.; Simonetti E. A. N.; Bastos T. M.; Cividanes L. S.; Thim G. P. Effects of octadecylamine functionalization of carbon nanotubes on dispersion, polarity, and mechanical properties of CNT/HDPE nanocomposites. J. Mater. Sci. 2018, 53 (20), 14311–14327. 10.1007/s10853-018-2627-3.

[ref15] Bao Y.; Pang H.; Xu L.; Cui C.-H.; Jiang X.; Yan D.-X.; Li Z.-M. Influence of surface polarity of carbon nanotubes on electric field induced aligned conductive network formation in a polymer melt. RSC Adv. 2013, 3 (46), 24185–24192. 10.1039/c3ra44356f.

[ref16] Benito P.; Herrero M.; Labajos F. M.; Rives V.; Royo C.; Latorre N.; Monzon A. Production of carbon nanotubes from methane: Use of Co-Zn-Al catalysts prepared by microwave-assisted synthesis. Chemical Engineering Journal 2009, 149 (1), 455–462. 10.1016/j.cej.2009.02.022.

[ref17] Wang J.; Shen B.; Lan M.; Kang D.; Wu C. Carbon nanotubes (CNTs) production from catalytic pyrolysis of waste plastics: The influence of catalyst and reaction pressure. Catal. Today 2020, 351, 50–57. 10.1016/j.cattod.2019.01.058.

[ref18] Wang Q.; Shang L.; Shi R.; Zhang X.; Waterhouse G. I. N.; Wu L.-Z.; Tung C.-H.; Zhang T. 3D carbon nanoframe scaffold-immobilized Ni3FeN nanoparticle electrocatalysts for rechargeable zinc-air batteries’ cathodes. Nano Energy 2017, 40, 382–389. 10.1016/j.nanoen.2017.08.040.

[ref19] Feng J.-M.; Dai Y.-J. Water-assisted growth of graphene on carbon nanotubes by the chemical vapor deposition method. Nanoscale 2013, 5 (10), 4422–4426. 10.1039/c3nr33855j. 23579565

[ref20] Atieh M. A.; Bakather O. Y.; Al-Tawbini B.; Bukhari A. A.; Abuilaiwi F. A.; Fettouhi M. B. Effect of Carboxylic Group Functionalized on Carbon Nanotubes Surface on the Removal of Lead From Water. Bioinorganic chemistry and applications 2010, 2010, 603978 10.1155/2010/603978. 21350599 PMC3038556

[ref21] Escobar M.; Moreno M. S.; Candal R. J.; Marchi M. C.; Caso A.; Polosecki P. I.; Rubiolo G. H.; Goyanes S. Synthesis of carbon nanotubes by CVD: Effect of acetylene pressure on nanotubes characteristics. Appl. Surf. Sci. 2007, 254 (1), 251–256. 10.1016/j.apsusc.2007.07.044.

[ref22] Song N.; Jia J.; Wang W.; Gao Y.; Zhao Y.; Chen Y. Green production of pristine graphene using fluid dynamic force in supercritical CO2. Chemical Engineering Journal 2016, 298, 198–205. 10.1016/j.cej.2016.04.022.

[ref23] Melvin G. J. H.; Ni Q.-Q.; Suzuki Y.; Natsuki T. Microwave-absorbing properties of silver nanoparticle/carbon nanotube hybrid nanocomposites. J. Mater. Sci. 2014, 49 (14), 5199–5207. 10.1007/s10853-014-8229-9.

[ref24] Ţucureanu V.; Matei A.; Avram A. M. FTIR Spectroscopy for Carbon Family Study. Critical Reviews in Analytical Chemistry 2016, 46 (6), 502–520. 10.1080/10408347.2016.1157013. 26941009

[ref25] Zhang X.; Zhou Y.; Mao Y.; Wei M.; Chu W.; Huang K. Rapid synthesis of ultrafine NiCo2O4 nanoparticles loaded carbon nanotubes for lithium ion battery anode materials. Chem. Phys. Lett. 2019, 715, 278–283. 10.1016/j.cplett.2018.11.053.

[ref26] Vennerberg D.; Hall R.; Kessler M. R. Supercritical carbon dioxide-assisted silanization of multi-walled carbon nanotubes and their effect on the thermo-mechanical properties of epoxy nanocomposites. Polymer 2014, 55 (16), 4156–4163. 10.1016/j.polymer.2014.06.020.

[ref27] Yang L.; May P. W.; Yin L.; Smith J. A.; Rosser K. N. Ultra fine carbon nitride nanocrystals synthesized by laser ablation in liquid solution. J. Nanopart. Res. 2007, 9 (6), 1181–1185. 10.1007/s11051-006-9192-4.

[ref28] Li J. P. H.; Zhou X.; Pang Y.; Zhu L.; Vovk E. I.; Cong L.; van Bavel A. P.; Li S.; Yang Y. Understanding of binding energy calibration in XPS of lanthanum oxide by in situ treatment. Phys. Chem. Chem. Phys. 2019, 21 (40), 22351–22358. 10.1039/C9CP04187G. 31576882

[ref29] Kowbel W.; Shan C. H. The mechanism of fiber—matrix interactions in carbon—carbon composites. Carbon 1990, 28 (2), 287–299. 10.1016/0008-6223(90)90003-H.

[ref30] Ganguly A.; Sharma S.; Papakonstantinou P.; Hamilton J. Probing the Thermal Deoxygenation of Graphene Oxide Using High-Resolution In Situ X-ray-Based Spectroscopies. J. Phys. Chem. C 2011, 115 (34), 17009–17019. 10.1021/jp203741y.

[ref31] Krishnan P.; Liu M.; Itty P. A.; Liu Z.; Rheinheimer V.; Zhang M.-H.; Monteiro P. J. M.; Yu L. E. Characterization of photocatalytic TiO2 powder under varied environments using near ambient pressure X-ray photoelectron spectroscopy. Sci. Rep. 2017, 7 (1), 43298 10.1038/srep43298. 28240300 PMC5327435

[ref32] Wei H.; Xia J.; Zhou W.; Zhou L.; Hussain G.; Li Q.; Ostrikov K. Adhesion and cohesion of epoxy-based industrial composite coatings. Composites Part B: Engineering 2020, 193, 108035 10.1016/j.compositesb.2020.108035.

[ref33] Dagdag O.; El Harfi A.; Essamri A.; El Gouri M.; Chraibi S.; Assouag M.; Benzidia B.; Hamed O.; Lgaz H.; Jodeh S. Phosphorous-based epoxy resin composition as an effective anticorrosive coating for steel. International Journal of Industrial Chemistry 2018, 9 (3), 231–240. 10.1007/s40090-018-0152-5.

[ref34] Neves J. C.; de Castro V. G.; Assis A. L. S.; Veiga A. G.; Rocco M. L. M.; Silva G. G. In-situ determination of amine/epoxy and carboxylic/epoxy exothermic heat of reaction on surface of modified carbon nanotubes and structural verification of covalent bond formation. Appl. Surf. Sci. 2018, 436, 495–504. 10.1016/j.apsusc.2017.12.031.

[ref35] Maity P.; Kasisomayajula S. V.; Parameswaran V.; Basu S.; Gupta N. Improvement in surface degradation properties of polymer composites due to pre-processed nanometric alumina fillers. IEEE Transactions on Dielectrics and Electrical Insulation 2008, 15 (1), 63–72. 10.1109/T-DEI.2008.4446737.

