# Supplementary material for: Application of Carbon Nanotubes from Waste Plastics As Filler to Epoxy Resin Composite
Source: ACS Sustain Chem Eng. 2022 Feb 1;10(6):2204–13. doi: 10.1021/acssuschemeng.1c07776 (PMC9097581; doi:10.1021/acssuschemeng.1c07776)
Supplement: Supplementary file 1 — sc1c07776_si_001.pdf [file sc1c07776_si_001.pdf]

SUPPORTING INFORMATION

Application of carbon nanotubes from waste plastics as filler to epoxy resin composite

Yuanyuan Wang †,‡, Ning Cai‡, Haiping Yang‡,*, Chunfei Wu†,*

†School of Chemistry and Chemical Engineering, Queen's University Belfast, Belfast, BT7 1NN, UK

‡ State Key Laboratory of Coal Combustion, School of Energy and Power Engineering, Huazhong University of Science and Technology, Wuhan, 430074, PR China

*Corresponding authors e-mail address:

Chunfei Wu: C.Wu@qub.ac.uk

Haiping Yang: Yhping2002@163.com

Number of pages: 5

Number of figures: 5

Number of tables: 1

Supporting figures

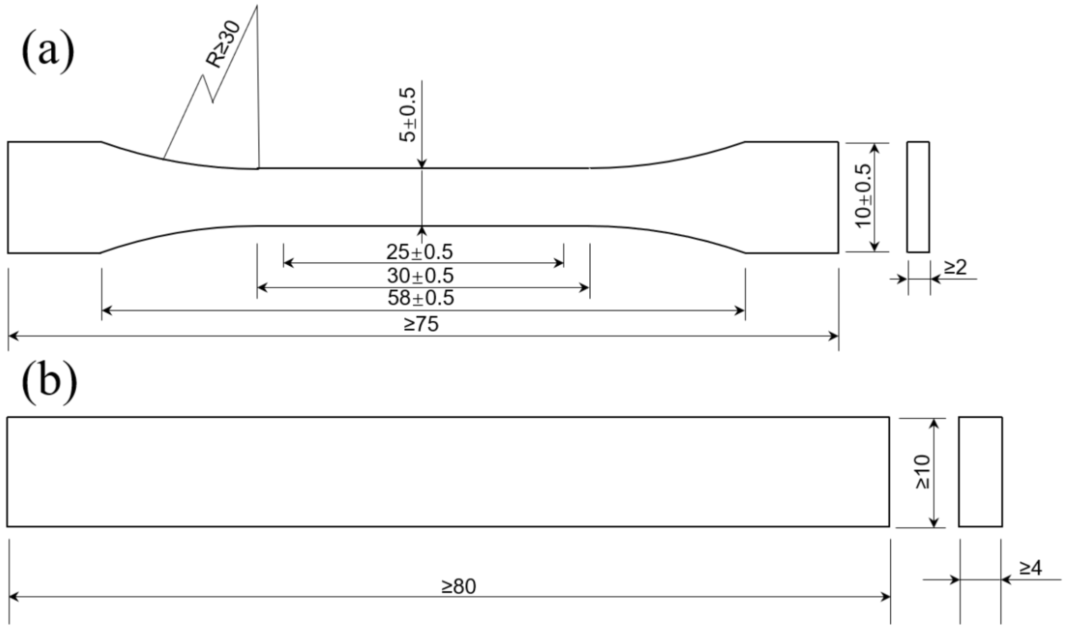

Figure S1. The size of specimens for (a) tensile and (b) flexural test.

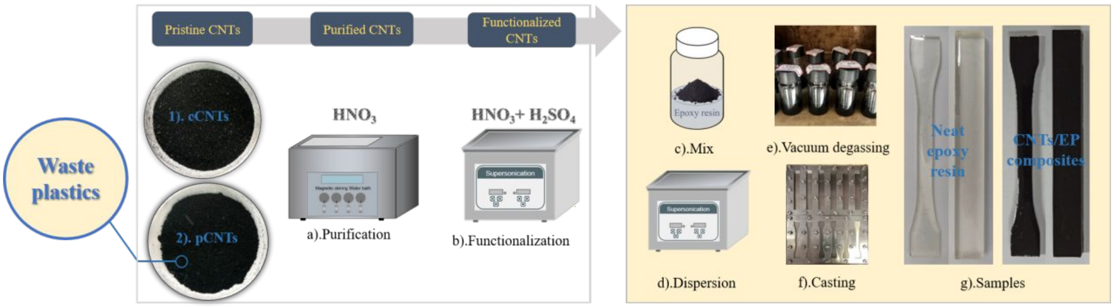

Figure S2. Schematic diagram of CNTs treatment and composite fabrication.

Supporting information

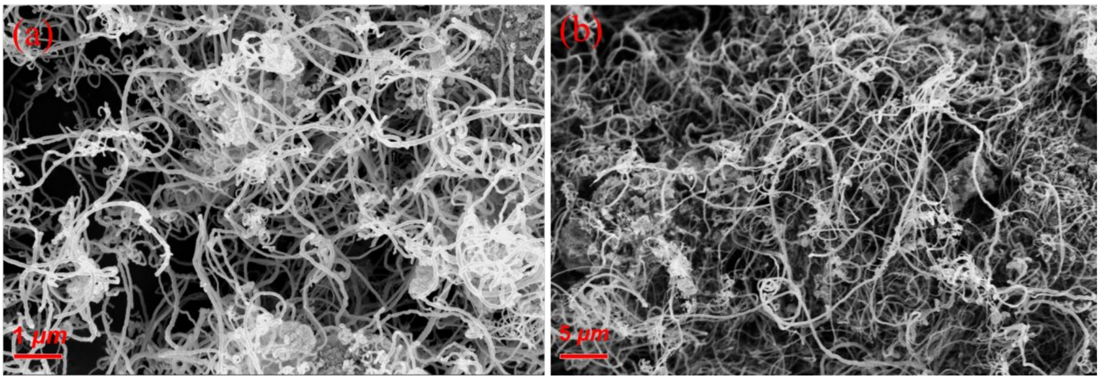

Figure S3. The length characterization of pCNTs: (a) TEM and (b) SEM results.

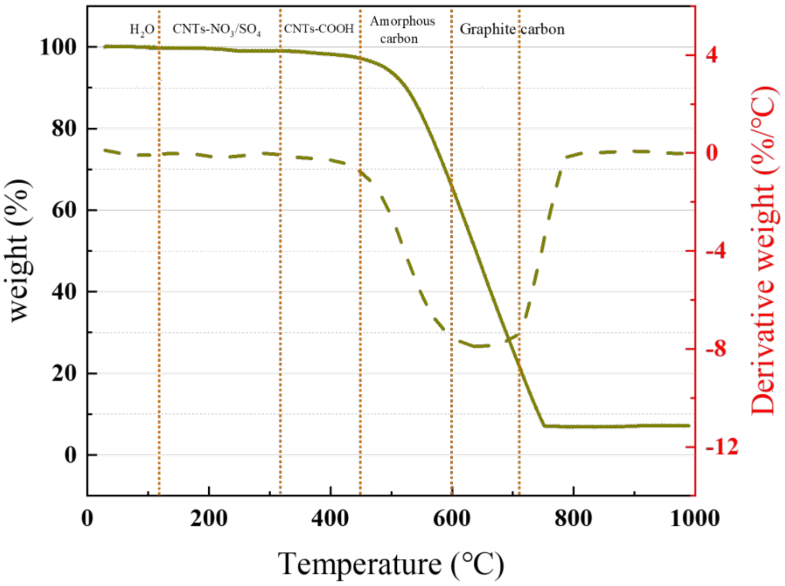

Figure S4. TGA thermograms of cMCNTs. (Possible proportions of Amorphous carbon, graphite carbon and metal are 35%, 58% and 7%, respectively.)

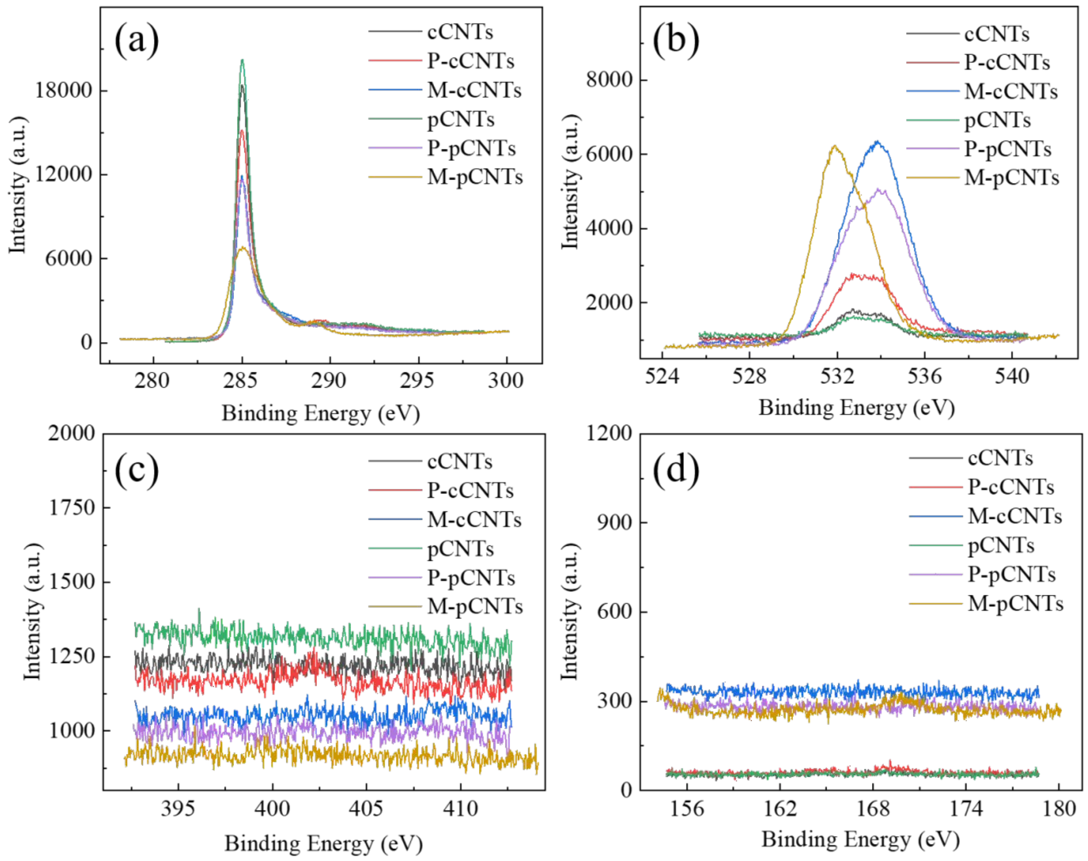

Figure S5. XPS figure of (a) C1s; (b) O1s; (c) N1s; (d) S1s spectra of different CNTs.

Supporting tables

Table S1. The composition of different CNTs/EP samples.

Name	CNTs	Resin content
	g	wt.%
Neat EP	0	100
0.1%CNTs/EP	0.007	99.90
0.25% CNTs/EP	0.018	99.75
0.5% CNTs/EP	0.035	99.50
1% CNTs/EP	0.700	90.91
2% CNTs/EP	1.400	83.33
6% CNTs/EP	4.200	62.50
